# Bulbar Onset Amyotrophic Lateral Sclerosis in a COVID-19 Patient: A Case Report

**DOI:** 10.7759/cureus.37814

**Published:** 2023-04-19

**Authors:** Mohammad Abu-Abaa, Aliaa Mousa, Sindhu Chadalawada, Ali Abdulsahib

**Affiliations:** 1 Internal Medicine, Capital Health Regional Medical Center, Trenton, USA

**Keywords:** dysphagia, dysarthria, bulbar palsy, amyotrophic lateral sclerosis, covid-19

## Abstract

Amyotrophic lateral sclerosis (ALS) is a devastating neurodegenerative disorder with a largely unknown etiology. In this case, we are presenting an 84-year-old male patient who was admitted for acute hypoxemic respiratory failure secondary to coronavirus disease 2019 (COVID-19) infection. He was neurologically intact. His infection improved and oxygen requirement was gradually weaned off allowing for discharge. However, he was admitted again a month later with progressive dysphagia and aspiration that were confirmed on videofluoroscopic study. He was also found to have mild dysarthria, bulbar muscle weakness, bilateral lower motor neuron facial nerve palsy, diffuse hyporeflexia on four extremities with intact sensory function. Diagnosis of ALS was suspected after extensive workup was pursued and ruled out nutritional, structural, autoimmune, infectious and inflammatory disorders. This case is only the third reported case in medical literature to suggest COVID-19 infection as a triggering/accelerating factor of ALS progression.

## Introduction

Amyotrophic lateral sclerosis (ALS) is a progressive neurodegenerative disorder that mainly affects the motor systems at levels of motor cortex, brainstem nuclei and anterior horn cells of the spinal cord. It usually starts focally and then spreads [1]. Although it was initially thought to be a pure motor disease, evidence of behavioral and language changes can be seen in up to 50% of cases. There is significant overlap between ALS and frontotemporal dementia (FTD) at molecular and neuropathological levels and they coexist in 15% of ALS cases [2]. According to the site of onset, ALS has been classified as spinal and bulbar ALS [3]. The relationship between ALS and coronavirus disease 2019 (COVID-19) infection is yet to be established in medical literature, hence the reason for this report. 

## Case presentation

**Figure 1:**
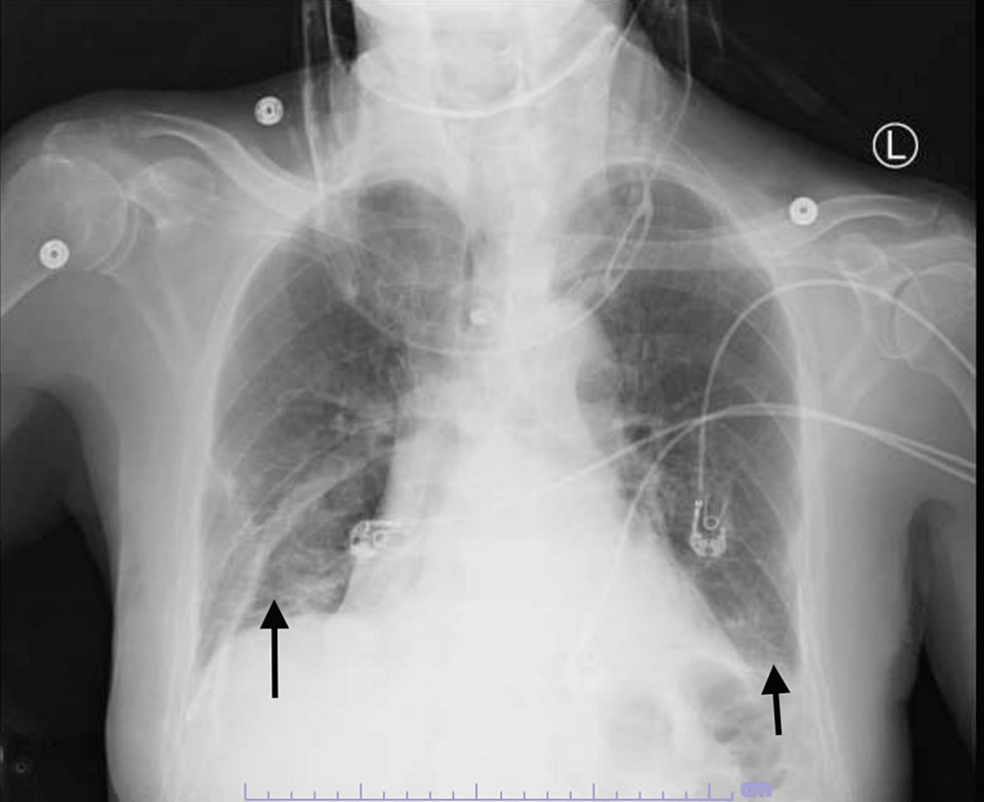
Chest X-Ray A chest x-ray showing evidence of bilateral basal opacities suggestive of COVID-19 pneumonia in the setting of acute hypodermic respiratory failure (arrows).

An 84-year-old male patient presented to the emergency department (ED) from a nursing home with a two days history of progressive dyspnea and dry cough. He also complained of bilateral upper and lower extremities tingling. Past medical history was significant for atrial fibrillation on apixaban and metoprolol, left traumatic below-knee amputation and recent fall that resulted in C4-C5 transverse process fracture that was stable and did not prompt surgical intervention. He also had received four doses of COVID-19 vaccines with the last dose given around a year ago. In ED, vitals signs included a temperature of 37.1 degrees Celsius, heart rate of 115 beats per minute with irregular rhythm, respiratory rate of 28 cycles per minute, blood pressure of 140/80 mmHg and oxygen saturation (SpO2) of 85% improving to 94% on 4 liters nasal cannula oxygen therapy. On the physical exam, he was alert, fully oriented, with C-collar, bilateral basal coarse inspiratory and expiratory crackles worse on the right side than the left. Neurological examination was grossly intact with Medical Research Council (MRC) muscle power of 5/5 all over, intact light touch and pinprick sensation grossly all over and intact cranial nerves examination. He tested positive for COVID-19 infection in the ED. Basic labs showed leukocytosis of 13,000 cells/ml, mild normochromic normocytic anemia of 13.4 g/dl, bandemia of 23%, elevated D dimer of 1.06 (reference 0-0.45 mcg/ml), elevated fibrinogen of 706 (reference 224-476 mg/dl), elevated C-reactive protein (CRP) of 21.5 mg/dl (reference less than 1) and hyponatremia of 128 mmol/L. Procalcitonin was normal and chest x-ray showed bilateral basal opacities (Figure 1). Patient was started on dexamethasone as well as remdesivir for a total of five days.

**Figure 2:**
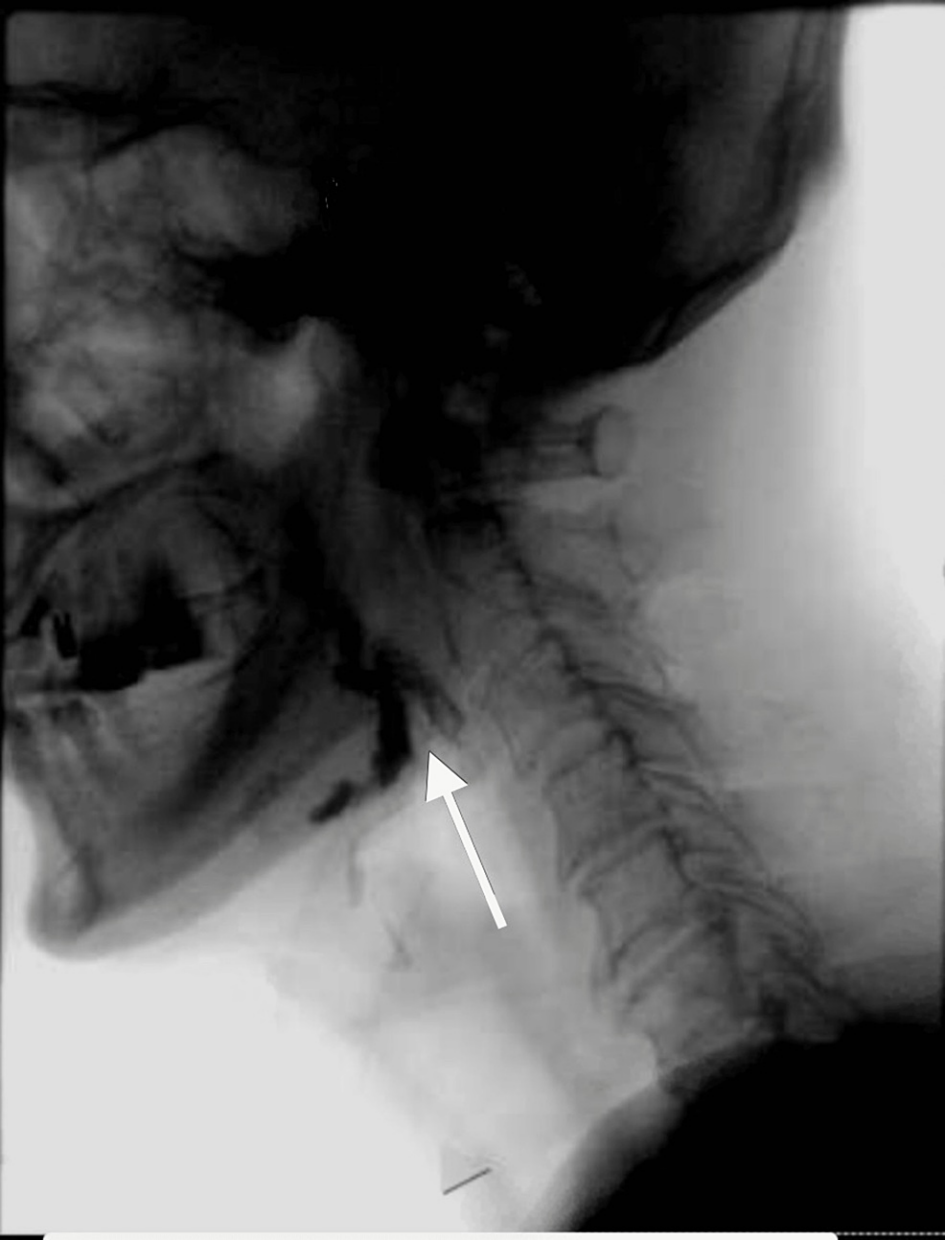
Video Swallow Study Videofluoroscopic swallow study showing aspiration as swallowed material spilled into the laryngeal vestibule prior to swallowing initiation (arrow).

**Figure 3:**
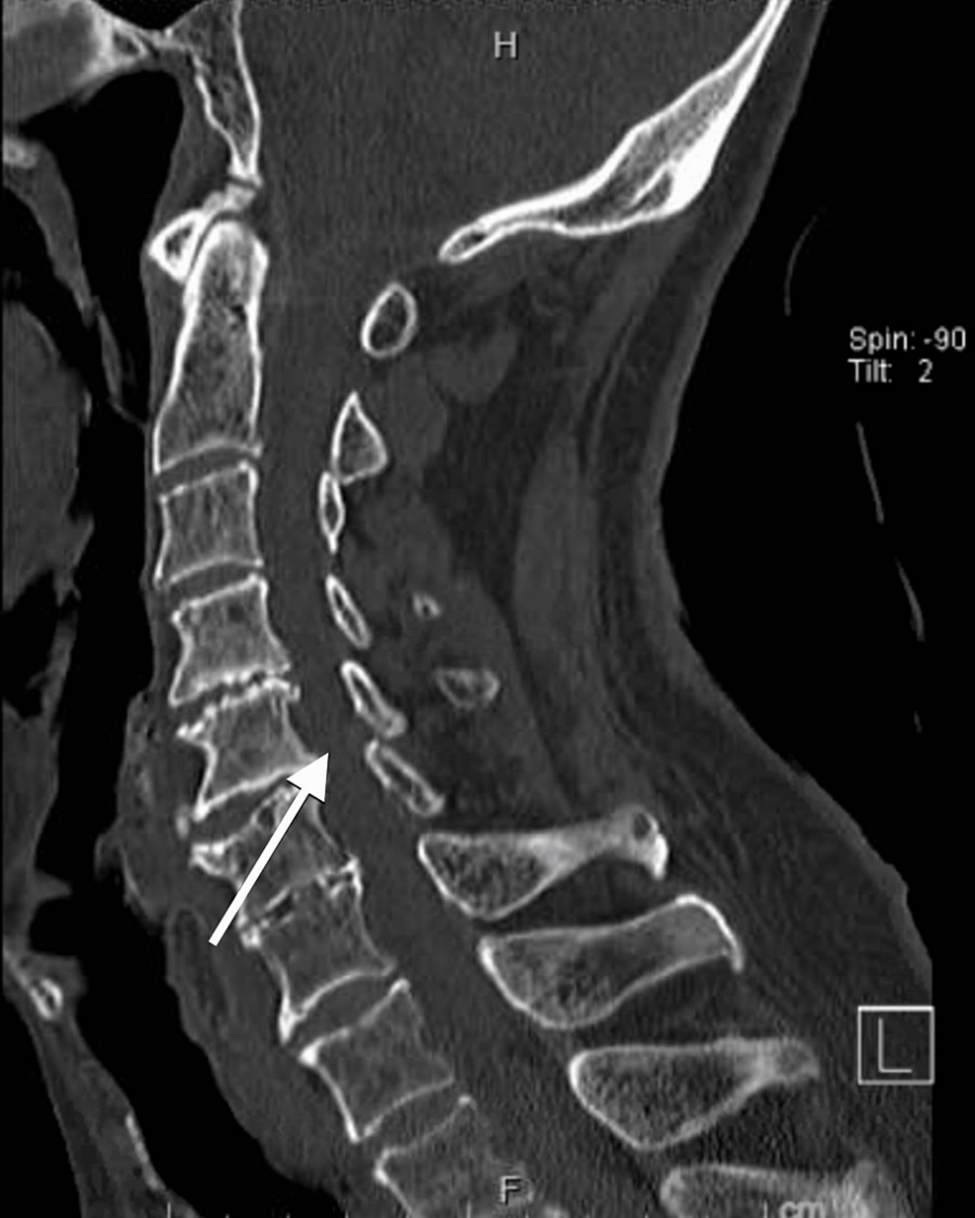
Computed Tomography of Cervical Spine Computed tomography (CT) scan of the cervical spine showing evidence of significant stenosis at C5-C6 level (arrow).

**Figure 4:**
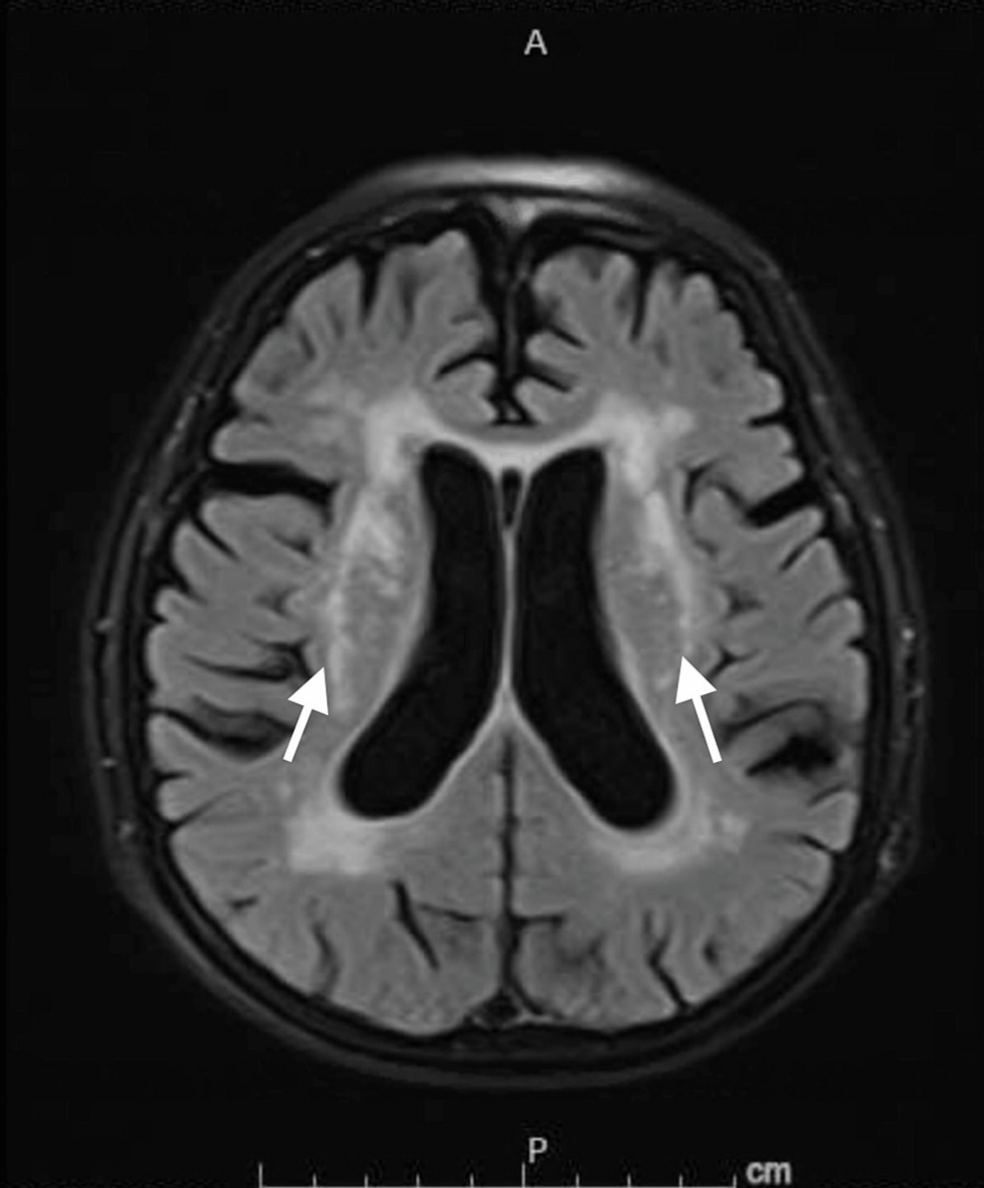
Magnetic Resonance Imaging of the Brain Magnetic resonance imaging (MRI) brain showing only bilateral periventricular old lacunae infarctions (arrows) with no other identifiable pathology explaining the clinical picture.

**Figure 5:**
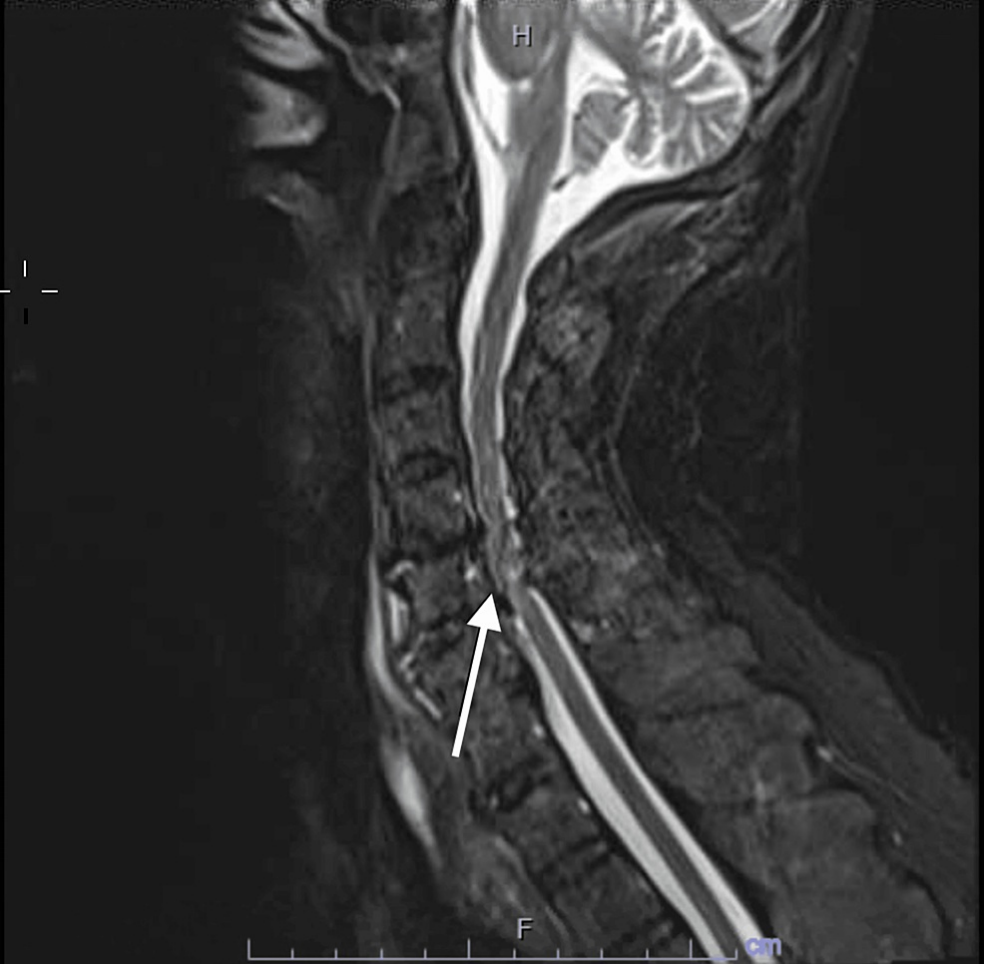
MRI Cervical Spine MRI cervical spine showing evidence of significant C5-C6 level spinal stenosis (arrow).

Due to his persistent cough, he was evaluated by speech and swallow evaluation service and demonstrated adequate and safe oropharyngeal swallow and bolus control with recommendation to keep the patient on a thin regular diet. Same recommendation was made on re-evaluation on day three of hospitalization. He did not complain of dysphagia. His oxygen requirement was weaned off and he was discharged back to his nursing home on room air after five days of hospitalization. However, it was noted that his cough was persistent and worsened by swallowing, which prompted re-evaluated by speech and swallow service one month later. He had a videofluoroscopic swallow study that showed aspiration as swallowed material spilled into the laryngeal vestibule prior to swallowing initiation (Figure 2). This prompted readmission of the patient where he was kept nil per mouth and started on parenteral nutrition. Physical exam showed fully oriented patient with bilateral masseter muscle wasting, mild dysarthria, weak elevation of the soft palate with no deviation to either side suggestive of bilateral symmetrical vagus and/or glossopharyngeal nerve palsy, bilateral asymmetrical hypoglossal nerve palsy as evidenced by difficulty protruding the tongue to either side and tongue muscle wasting with no apparent fasciculation, bilateral upper motor neuron facial nerve palsy as evidenced by difficulty puffing out the cheeks but otherwise intact cranial nerves sensory and motor examination. Further examination showed muscle power MRC 4/5 on bilateral upper extremities and lower extremities, hyporeflexia with +1 deep tendon reflexes all over the four extremities with intact gross sensory function all over the body. Computed tomography (CT) of head as well as cervical spine were unremarkable except for mild spinal canal stenosis at the C5-6 level (Figure 3). Magnetic resonance imaging (MRI) was remarkable only for chronic lacunes (Figure 4). MR angiography (MRA) of the brain and neck ruled out any vascular abnormality. Serum levels of folate, vitamin E and B12 were within normal limits. Creatine kinase (CK) level was also normal. MRI of cervical spine showed significant stenosis at C5-6 (Figure 5). He had lumbar puncture (LP) and cerebrospinal fluid (CSF) analysis that showed clear fluid with lymphocytic pleocytosis of 9 cells/ml, and normal glucose and protein levels. CSF culture as well as meningitis/encephalitis panel were negative.

Autoimmune neuromuscular panel including acetylcholine receptor binding, blocking, and modulating antibodies, ganglionic acetylcholine receptor antibodies, P/Q-type and N-type voltage-gated calcium channels, voltage-gated potassium channels, titin antibody, striated muscle antibodies, and leucine-rich glioma-inactivated protein 1 antibody and contactin-associated protein-2 antibody IgG in addition to anti-Musk antibody were negative. Antiganglioside antibodies including GM1, GM2, GD1a, GD1b, and GQ1b were negative. The patient was started empirically on intravenous immunoglobulin (IVIG) for five days. No clinical improvement nor improvement in dysphagia on repeat videofluoroscopic study was appreciated after complete IVIG therapy. The patient had evidence of motor axonal loss on nerve conduction study affecting the left median, ulnar, fibular and tibial nerves. There was also reduced combined muscle action potential (CMAP) amplitude and slowed conduction velocity affecting the right median, tibial and fibular nerves with relative preservation of sensory nerve action potential (SNAP) in the upper extremities. Electromyography (EMG) was largely limited by technical difficulties as well as scarring from previous trauma of the right lower extremity but showed +2 sharps and fast firing units. Clinical progression was evident with progressive bilateral facial muscle weakness and wasting, progressive weakness of palatal elevation and occurrence of tongue fasciculations. The patient had placement of a percutaneous endoscopic gastrostomy tube and was referred to ALS center.

## Discussion

ALS has an incidence rate of 1.75-3 per 100,000 per year, which increases to 4-8 per 100,000 per year in the age group between 45-75 years [4]. The typical age of onset is usually 58-63 years in sporadic ALS and 40-60 years in familial ALS [4]. The incidence of ALS is slightly higher in males than females, and males are more likely to develop sporadic limb onset type of ALS than females [5,6]. Differential diagnoses of ALS in this case included myasthenia gravis, Gillian Barres syndrome, Miller Fischer syndrome and autoimmune demyelinating disorders. Although the speed of progression was atypical for ALS, the diagnosis of ALS in this particular case was suggested by EMG and nerve conduction study (NCS) as well as ruling out other explaining etiologies based on negative serology and lack of response to empiric IVIG. 

Heterogeneity of the disease is seen not only at the clinical level but also at genetic and neuropathological levels [7]. The clinical cornerstone of ALS presentation is adult onset muscle weakness and wasting that is progressive in nature [1]. The most common type of ALS is spinal ALS, which is seen in two-thirds of cases and is characterized by distal upper or lower limb onset of muscle weakness and atrophy. Bulbar ALS is seen only in one-third of cases and is characterized by bulbar onset of symptoms including dysphagia, dysarthria, dysphonia, and reduced mouth closure and chewing problem. Pseudobulbar palsy with emotional lability can also be seen [3]. The diagnosis of ALS is based on history, physical exam, and EMG as well as neuroimaging to rule out other pathologies [8]. 

The pathogenesis of ALS is thought to be a combination of genetic, environmental and age-related factors, where genetic factors account for 30-60% of the susceptibility to develop ALS [5]. Multiple genetic mutations have been linked to sporadic and familial cases of ALS, the most common of which is hexanucleotide repeat expansion in the C9orf72 gene which is seen in 30-50% of familial and 7% of sporadic ALS [1]. Multiple environmental risk factors have also been reported, although with no established causal relationship, including smoking, exposure to metals and pesticides, head injury and viral infections [1]. Enteroviruses including poliovirus, echovirus, coxsackievirus, and enterovirus, have been suggested as risk/causal factors for development of sporadic ALS through induction of molecular changes and establishment of a persistent infection in the central nervous system (CNS) [9,10]. Similarly, it was recently reported that certain viruses including human immunodeficiency virus type 1 (HIV-1), Zika virus and human rabies virus can exacerbate the molecular changes characteristic of ALS and hence exacerbate the clinical progression of ALS, establishing a possible risk/causal factor relationship [11]. 

In our patient, the suggestion of ALS was based on bulbar symptoms and EMG findings. It is likely that cervical spinal stenosis and body degenerative changes were incidental and cannot explain his bulbar dysfunction. However, the temporal relationship between COVID-19 and ALS raises the concern of possible relationship. Also, the rapid progression of initial bulbar involvement that progressed within less than a month to involve facial nerves was suggestive of accelerated progression. Two cases of accelerated ALS progression by COVID-19 infection were reported [12]. In both of these cases, ALS diagnosis was established several years prior to COVID-19 infection. A retrospective cohort study in France involving 84 patients with established diagnosis of ALS were noted to have worsened functional decline during the pandemic lockdown, although it was not reported that any had COVID-19 infection and this effect was postulated secondary to decreased medical contact rather than COVID-19 itself [13]. In contrast, our patient had no prior diagnosis or clinical manifestation suggestive of ALS prior to COVID-19 infection. This raises the suspicion of COVID-19 infection as a trigger vs progression accelerator of underlying neurodegenerative disorders.

A recent observational study examined the association between COVID-19 infection and neurodegenerative disorders. It showed a significant association between COVID-19 infection and Alzheimer’s disease. However, it failed to show association with other degenerative disorders including ALS, which might be attributable to sample size [14]. The suggested mechanisms include likely neuroinvasion, neuroinflammation and blood-brain barrier dysfunction [15]. 

## Conclusions

To the best of our knowledge, this is the third case reported of a possible relationship between COVID-19 infection and ALS. However, unlike the previous reported cases, this case had the clinical onset of the disorder shortly after COVID-19 infection. As other viral infections have been previously suggested to play a role in ALS pathogenesis with similar molecular changes and COVID-19 is becoming increasingly recognized to have neurotropism, it seems plausible to raise the concern of this yet-to-be-proven relationship.
